# Three-Dimensional Simulation of the Temperature Distribution in a Commercial Broiler House

**DOI:** 10.3390/ani12101278

**Published:** 2022-05-17

**Authors:** Patrícia Ferreira Ponciano Ferraz, Ednilton Tavares de Andrade, Regina Batista Vilas Boas, Renan Pereira Rezende, Tadayuki Yanagi Junior, Matteo Barbari

**Affiliations:** 1 Department of Agricultural Engineering, Federal University of Lavras (UFLA), Lavras 37200-900, Brazil; ednilton@ufla.br (E.T.d.A.); regina_lavras@yahoo.com.br (R.B.V.B.); renanprezende@gmail.com (R.P.R.); yanagi@ufla.br (T.Y.J.); 2 Department of Agriculture, Food, Environment and Forestry, University of Firenze, 13-50145 Firenze, Italy; matteo.barbari@unifi.it

**Keywords:** broiler farming, thermal comfort, computational fluid dynamics, welfare

## Abstract

**Simple Summary:**

Suitable environmental conditions in broiler houses are essential for animal welfare and successful broiler production. Animals’ effective adaptations to environmental challenges are crucial to their development and production. Computational fluid dynamics (CFD) tools can be seen as an alternative to evaluating indoor environmental conditions. The primary aim of this paper was to evaluate the environment inside a commercial broiler house provided with a heating system. The proposed CFD model presented a good simulation of the experimental data. This analysis indicated the existence of failures in the heating system in some areas of the broiler house during the experimental periods, and it may cause discomfort for the broilers and consequently affect the chicks’ productive and economic losses. Thus, these obtained results can be used to drive decision-making processes to create satisfactory environmental conditions for the development of chicks.

**Abstract:**

The aim of this paper was to analyze, using computational fluid dynamics (CFD), a heating system in a commercial broiler house. Data were collected in a broiler house located in the western mesoregion of Minas Gerais, Brazil. The data were collected at 10 a.m. on the seventh day of chicks’ life in 16 points inside the house. A tetrahedral mesh was adopted for the simulation, and testing of the mesh yielded a geometry of 485,691 nodes. The proposed model was developed in a permanent state condition to simulate the temperature air inside the broiler house, and all other input variables were considered constant. The applied CFD technique resulted in satisfactory fitting of the air temperature variable along the broiler facility as a function of the input data. The results indicated that the model predicted the environmental conditions inside the broiler house very accurately. The mean error of the CFD model was 1.49%, indicating that the model is effective and therefore that it can be used in other applications. The results showed that the heating system provided favorable thermoneutral conditions for chicks in the biggest part of the broiler house. However, there were some areas with air temperature above and below the thermoneutral zone

## 1. Introduction

One of the most essential factors in improving broiler production is providing an appropriate environment inside the broiler house (air temperature, relative humidity, air velocity, air quality, and gases) with lower possible costs [1]. Microclimatic conditions in broiler rooms depend on the temperature and humidity of the air, lighting, ventilation, as well as concentration of harmful gases [2]. As mentioned, several factors can influence broiler development, but thermal stress is one of the most responsible environmental factors influencing a wide range of broilers performances, including animal welfare and reduced feed intake, which in turn affect feed conversion ratio, growth rate, body weight, meat quality, and others [3]. These negative influences on the poultry system may result in significant economic losses.

In the first days of life, chicks are very sensitive to different comfort conditions, and air temperature can be considered the environmental factor with the greatest impact on broiler development because it affects homeothermy [4]. In the firsts week of life, chicks present a fast metabolism and growth rate. These animals have a poor ability to adjust to the thermal environment fluctuations [5]. Therefore, the thermal stress in the early development of broiler chickens exerts a very negative effect on the animals through physiological and behavioral mechanisms [6]. According to [7], the first days of broiler life are the most critical, and errors made in this phase cannot be satisfactorily corrected in the future, thus it can affect the final broiler development and performance. Yearly chicks do not have sweat glands, and they are highly sensitive to heat stress [5], and due to their fast growth, commercial broiler chicks are particularly susceptible to climatic challenges.

It is important to mention that the heating systems used in tropical environments, particularly in opened-sided broiler houses, generally do not produce constant temperatures, which can cause developmental losses and even lead to death in extreme cases [8]. Additionally, the heating systems can have an economical importance because it can affect the energy requirements of buildings [9].

To improve the broiler production, the creation of appropriate thermal conditions inside the broiler house is one of the biggest challenges. The evaluation of the internal environment is mandatory for explaining its destructive impacts in the broiler production [10]. The poultry industry is continuously working to reduce the effects of the parameters inside the house, such as temperature, humidity, air velocity, gases, and others, on the animal welfare. We can consider that all of these mentioned parameters are governed by airflow patterns. Thus, it is important to understand the principles of air movement to provide the correct quantities of air and the proper distribution patterns to meet the needs of the broiler house [11].

However, due to the complexity of the phenomena involved in the internal thermal environment of broiler houses, field experiments can be very difficult [12]. The amount of information required to quantify the environmental variables entirely depends on the physics involved and the level of precision associated with the analysis tools [11]. Therefore, Computational Fluid Dynamics (CFD) techniques can be used as an alternative to determine the environmental conditions inside the broiler facility. The CFD method allows solving numerically difficult, long, and complex equations through a computer and analyzing the distribution of desired parameters associated with the flow [13]. According to [11], CFD can efficiently develop both spatial and temporal field solutions of fluid pressure, temperature, and velocity, proving its effectiveness in system design and optimization. Given this context, CFD models have been used to carry out projects that can improve broiler houses. Although there are few works carried out in CFD applied to the understanding of the internal environment of broiler facilities, the existing ones demonstrate the advantages of this technique to deepen the studies of heat and mass transfer phenomena, as well as to improve and optimize the design of the building with the aim to obtain the best animal thermal comfort, finding the best combinations in the use of natural and mechanical ventilation, and evaporative cooling systems [14].

This research aimed to evaluate the thermal conditions in a broiler house heated by an industrial metal furnace using the CFD technique to characterize the air temperature and its distribution profiles inside the house as a whole and thus to improve and optimize broiler thermal well-being in existing facilities.

## 2. Materials and Methods

**Figure 1:**
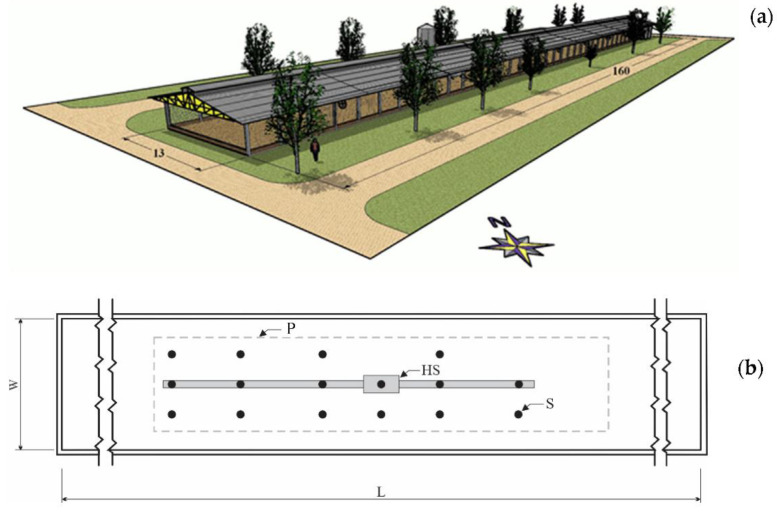
(**a**) Diagram of the broiler house evaluated in this study, indicating the main dimensions in meters. (**b**) Scheme of the position of sensors/recorders of air temperature, where W is the width of the area available for the broiler chicks and L is the length, HS is the heater system, and P is the plywood sheet.

The experiment was carried out in a commercial broiler farm in the western mesoregion of Minas Gerais, Brazil during the spring season. The farm is located at the mean geographic coordinates of 20°11′58′′ south latitude and 45°02′08′′ west longitude. The studied broiler house was 13 m in width, 160 m in length, and 3 m floor-to-ceiling eaves height (Figure 1a). The floor was concrete covered with rice husk bedding. The exterior walls of the house had 0.35 m of height and double curtains (one internal and one external) made of yellow plastic tarpaulin, placed 2.45 m from the ground.

The internal area of the broiler house was bordered with plywood sheets with a length of 54.3 m, width of 8.0 m, and height of 0.6 m so that the chicks were as close as possible to the heating systems (Figure 1b). The environmental heating system used consisted of an industrial metal furnace with indirect biomass burning, with a length of 2.23 m, width of 1.23 m, and height of 1.85 m. The heated air was blown by a three-phase motor with 2206 W of power, 1725 RPM, through approximately 28.6 m of metal tubing on the northeast side and 22.45 m on the southwest side, installed in the central inner part of the house. The tubing had a diameter of 0.23 m and holes 0.05 m in diameter separated by 1.0 m located alternately on each side to distribute the heated air. During the experimental period, the ventilation system was turn off.

A total of 28,000 7-day-old male Cobb chicks were used to perform this study. On the seventh day of life, the chicks were distributed in the density of 54 poultry m^2^. To characterize the thermal environment, measurements of the air temperature inside the broiler house were taken by sensors/recorders Hobo Pro Series—Onset^®^ (reading accuracy of ± 3%) (in 16 points), as shown in Figure 1b.

The data were collected when the chicks were seven days of life at 10 a.m. The measurements were performed at a height compatible with the area occupied by the chicks at 0.10 m from the litter, as recommended by [15].

**Figure 2:**
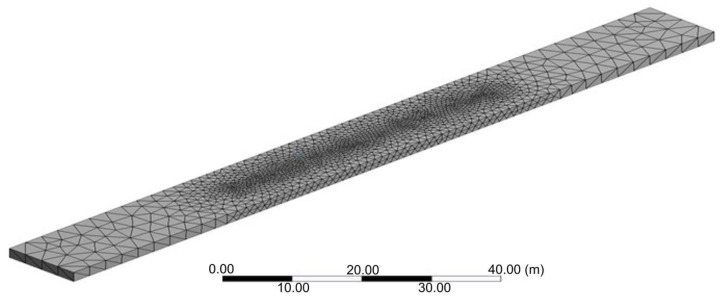
Structural representation of the tetrahedral mesh of the studied broiler house with 13 m in width, 160 m in length.

To create the 3D geometry of the interior of the broiler house, a computational domain was created using [16] and then transferred to the software [17] for the development of a mesh. The geometry of the building was modelled following its real dimensions. Different types of tetrahedral and quadratic meshes were generated aiming at optimum results, but the best results were obtained using tetrahedral mesh. According to [18], this mesh presents more favorable results for the study object. After the computational mesh test, the use of a tetrahedral mesh with 485,691 nodes was defined (Figure 2).

### Conservation Equations

The equations used for the simulation were based on the Navier–Stokes equations, i.e., mass, momentum, and energy (conservation equations). Equation (1) is known as the momentum equation and represents the principle of mass conservation. Equation (2) represents the general characteristic of mass equations; that is, the temporal variation in the fluid is equal to the resultant force acting on it.

(1) $$\frac{\partial \rho }{\partial t}+\frac{\partial }{\partial x_{i}}\cdot \left(\rho u_{i}\right)=0$$

(2) $$\frac{\partial }{\partial t}\left(\rho u_{i}\right)+\frac{\partial }{\partial x_{j}}\left(\rho u_{i}u_{j}\right)=-\frac{\partial }{\partial x_{i}}P+\frac{\partial }{\partial x_{j}}\tau_{ij}+\rho g_{i}+F_{i}$$

where

ρ—fluid density (kg m^−3^);
t—time (s);
$x,x_{i},x_{j}$—length of the components (m);
$u_{i},u_{j}$—velocity of the components (m s^−1^);
*P*—pressure (Pa);
$\tau_{ij}$—tension (Pa);
$g_{i}$—gravitational acceleration (m s^−2^); and
$F_{i}$—direction of external forces (N m^−3^).

The heat generated by the chicks in the CFD simulation was calculated as follows Equations (3) and (4). These equations were added to the model simulating the heat produced by the chicks.

A = 8.19 w^0.705^ (3)

where

A—approximate bird area (m^2^);
w—body weight (g). It was considered the body weight of 194 g according to [19].

Produced heat Equation (4) [20]

Qt = 8.09 w^0.75^ (4)

where

Qt—heat produced (W);
w—body weight (kg).

The proposed model was developed in a permanent state condition. The CFD simulation considered only the air temperature leaving the heating duct. The heating system provided a flow of 6800 m^3^ h^—1^ at 54 distribution points inside the broiler house. The CFD simulation did not represent the ventilation system because it was turned off during the experiment period. All other input variables such as environment outside, animals (age, weight, density), relative humidity, and ventilation were considered constant. Additionally, the following simplifications were made for developing the CFD simulation: drinkers, feeders, litter roughness and thickness, and lights were neglected.

The results obtained by the CFD simulation were compared with the data obtained experimentally in the field for the validation of the proposed model. A total sample of 16 experimental data was taken and compared with the CFD results.

To analyze the representative nature of the three-dimensional simulation of the temperature distribution in a commercial broiler house, we compared the experimental data with the values estimated for model, checking the mean relative percentage of error (P), mean estimated error (SE), and chi-square test (χ^2^), according to the Equations (5)–(7), respectively [21].

(5) $$P=\frac{100}{n}\sum \frac{\left|{Y-Y}_{0}\right|}{Y}\;$$

(6) $$\mathrm{SE}=\sqrt{\sum \frac{{\left({Y-Y}_{0}\right)}^{2}}{\mathrm{DF}}}$$

(7) $$\chi 2=\sum \frac{{\left({Y-Y}_{0}\right)}^{2}}{\mathrm{DF}}$$

in which,

Y—value observed experimentally, dimensionless;
Y_0_—value calculated by the model, dimensionless;
n—number of experimental observations;
DF—degrees of freedom of the model.

## 3. Results

**Figure 3:**
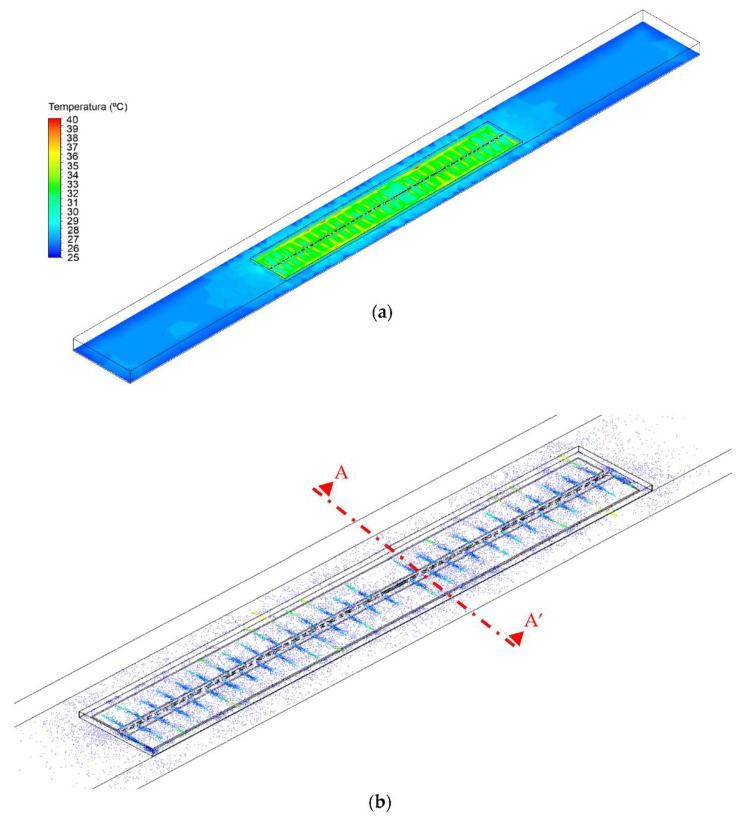
Three-dimensional representation of (**a**) Air temperature distribution inside the broiler house at 0.10 m height and (**b**) air flow distributions inside the studied broiler house with 13 m in width, 160 m in length. Red line indicates the position of the section AA′.

**Figure 4:**
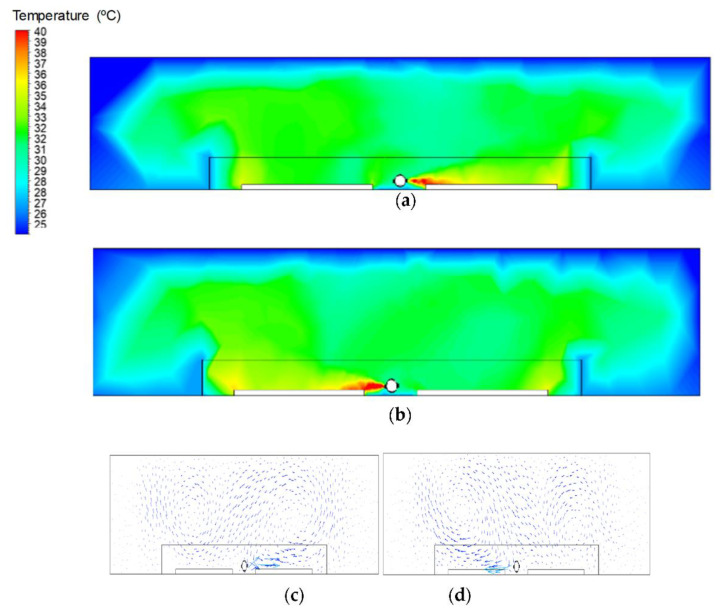
Perspective of the airflow distribution on right (**a**,**c**) and left (**b**,**d**) sides of the hot air outlet duct and inside the broiler house. The indication of position of the seccion in the broiler house is indicated by AA′ lines on Figure 3b.

As illustrated in Figure 3 and Figure 4, the CFD simulation determined the air temperature distribution and the air flow inside the broiler house heated by an industrial heater during the seventh day of chicks’ life at 10 a.m.

Figure 3 shows the top view of the entire area of the broiler house and the internal area bordered with plywood sheets. This internal area is indicated by the black rectangle in the middle of the house. This delimited area is the chicks’ zone, and it is important to keep the animals as close as possible to the heating systems.

At the ends of the broiler house, around the area occupied by the chicks, there is a trend toward lower temperatures indicated by the blue area. However, in the area occupied by chicks, the air temperature distribution is also not homogeneous. As shown in Figure 3a, there was a clear discrepancy between the air temperature close to the heating system pipe openings and the rest of the house. The air temperature in the broiler zone ranged between 26.7 (light blue) and 40 °C (red). The lowest temperature was in the middle of the house where the heater system structure was located, and the highest temperature was reached where the heating system pipe openings were placed. It is possible to observe that the hot air coming out of the heater pipe reaches the internal plywood sheets and returns to the chick’s zone. The uneven distribution of air temperature inside the house can generate regions with inadequate thermal conditions for the development of chicks. This heterogeneity of the air temperature inside the chicks zone could affect the average feed conversion ratio, growth rate, body weight, the development of the animals, and the costs of production consequently. Appropriate environmental conditions during the first week of life are very important [22].

Figure 3b indicates that the air flows homogeneously throughout the environment, with no concentration of air at any point. It can collaborate with the air temperature distribution in the broiler house. The homogeneity in the air flow distribution in the zone occupied by the chicks is important to inhibit migration into more comfortable but crowded regions [23].

Figure 4a,b show the air temperature distribution in the floor plan section view of the broiler house. The position of the section is indicated in Figure 3b by the red lines. It is possible to observe the hot air temperature distribution at the outlet of each side of the heating system inside the chick occupation area. The majority of the house is under the thermoneutral temperature for chicks, around 32 °C [15] indicated by the green area. However, the temperature of the hot air leaving the heating system tubing is greater than 39 °C. In addition, the figures also show some unfavorable conditions, with a mean air temperature greater than 36 °C represented by the yellowish areas near the plywood sheets, indicating unfavorable thermal comfort conditions in the first week of life of the chicks. According to [24], during the initial life period of the chicks, the control of the thermal variables inside the broiler facility must be better. When the chicks are submitted to uncomfortable thermal conditions, it may compromise the thermoregulatory system development and can cause respiratory diseases. According to [4], it is essential to emphasize that submitting young chicks to thermal challenge, even for a small period, may affect their growth, development, and welfare, so they may not be able to recover adequately. For this reason, it is very important to maintain an appropriate microclimate inside the poultry facility and to improve the animal welfare and production ultimately.

Figure 4c,d show that both the left and right hot air outlets provide a homogeneous and satisfactory lateral distribution of air flow throughout the environment. The air flow rate was considered uniform in all heated air outlets, and the value of 6800 m^3^ h^−1^ was adopted.

### Validation of the Model

The results obtained from the CFD simulations were verified and compared with the corresponding data of air temperature obtained experimentally with a sample size of 16 experimental measurements.

As shown in Table 1, the results obtained indicate the good accuracy of the model in predicting the environmental conditions inside the broiler house. According to these results, the simulated air temperature values were similar to those that were observed experimentally. The CFD model exhibited a mean relative percentage of error (P) of 1.49, mean estimated error (SE) of 1.35, and chi-square test (χ^2^) of 1.81, indicating that the CFD system satisfactorily simulates the temperature values. According to [25], results can be considered satisfactory for error less than 20%. A CFD model to simulate the summer and winter indoor environments in a mechanically ventilated broiler house were developed by [26]. These authors have considered the relative error (%) to simulate the temperature between of −5% or 5% in winter and summer as a good result. [27] studied the thermal environment inside of a poultry house based on temperature and airspeed measurements, and they have found most of the error values between −2.50% or 2.50% to predicted temperature.

Based on the obtained results in this research, it was concluded that the proposed CFD simulation could be used to characterize the air temperature distribution inside the broiler facility adequately. The tested model can be used to improve the evaluation of the indoor environment, the placement of heater systems, and the architectural design of the structures.

According to [28], the use of environmental variables to evaluate the thermal condition inside a broiler house allows determining the comfort/discomfort situation of the broiler due to situations that are adverse to the thermal comfort zone recommended by the literature. Understanding the distribution of air temperature inside a broiler house can be used to improve thermal comfort of the animals and improve the design of the project [18]. As well as improvements in the heating system such as more homogeneous and less turbulent heated air outlets, providing better mixing of the heated air with the ambient air.

This analysis allowed observing some failures in the heating system in some areas of the broiler house during the experimental period. These failures may affect the chick’s welfare, cause discomfort to the animals, and cause productive and economic losses. Thus, obtained results can be useful to drive decision-making processes aimed at creating appropriate environmental conditions for chicks. At the same time, this can improve the environment in the broiler house, with the aim of achieving thermal comfort for the broilers, saving energy, and improving efficiency. These advantages can contribute to animal welfare, productivity, and environmental sustainability [18].

## 4. Conclusions

In this study, the thermal conditions in a broiler house heated by an industrial metal furnace were analyzed. The proposed CFD model presented a good simulation of the experimental data in a permanent state condition and can be used to predict patterns of air temperature inside a broiler house. Additionally, it can simulate the heating system of other broiler houses with a similar heating system in a satisfactory way. With this, it is possible to optimize these systems, obtaining more adequate conditions to guarantee the thermal comfort of the birds with savings of resources that would be spent with physical modifications of the facility and more efficient constructions.

Based on the images, it is concluded that the biggest part of the studied house was in a comfortable temperature for chicks, but there were some regions with air temperature above and below the thermoneutral zone. These failures in the heating system indicated by the results may cause discomfort to the animals giving productive and economic losses.

## Figures and Tables

**Table 1:** Comparison of the air temperature values (°C) inside the broiler house obtained experimentally and simulated by the model.

	X	Y	Experimental Air Temperature (°C)	Simulated Air Temperature (°C)	Mean Error
1	110.7	6.5	28.7	28.45	0.87
2	110.7	3.0	27.5	27.45	0.18
3	73.4	3.0	29.3	28.75	1.88
4	73.4	6.5	28.2	27.55	2.30
5	73.4	9.9	29.9	29.25	2.17
6	99.2	9.9	28.7	28.55	0.52
7	99.2	6.5	28.3	28.55	0.88
8	99.2	3.0	28.6	28.55	0.17
9	86.4	6.5	29.4	29.45	0.17
10	86.4	3.0	29.8	29.15	2.18
11	60.3	9.9	28.9	28.55	1.21
12	60.3	6.5	29.9	29.55	1.17
13	60.3	3.0	28.9	29.15	0.87
14	48.5	9.9	28.9	28.05	2.94
15	48.5	6.5	28.7	27.95	2.61
16	48.5	3.0	26.7	27.25	2.06
				P (%)	1.49
				SE (decimal)	1.35
				χ^2^ (decimal)	1.81

## Data Availability

The data presented in this study are available on request from the corresponding author.
